# LncRNA ZFHX4-AS1 initiates an oncogenic axis involving a ZFHX4/SOX2 positive feedback loop to accelerate glioma progression

**DOI:** 10.3389/fonc.2025.1687984

**Published:** 2025-12-08

**Authors:** Hongshan Yan, Xue Wang, Zongmao Zhao

**Affiliations:** 1 Department of Neurology, The First Hospital of Hebei Medical University, Shijiazhuang, China; 2 Department of Neurosurgery, The Second Hospital of Hebei Medical University, Shijiazhuang, China

**Keywords:** long non-coding RNA, ZFHX4-AS1, ZFHX4, SOX2, glioma

## Abstract

**Introduction:**

The long non-coding RNA ZFHX4-AS1 is a recently identified transcript with an unknown role in glioma. Here, we demonstrate that ZFHX4-AS1 and its neighboring protein-coding gene, ZFHX4, are both significantly upregulated in glioma, and their high expression correlates with poor patient prognosis.

**Methods:**

We integrated pan-cancer and glioma transcriptomic datasets from TCGA to assess ZFHX4-AS1 and ZFHX4 expression patterns and their prognostic relevance. We analyzed the expression of ZFHX4-AS1 and its neighboring gene ZFHX4 in human glioma tissues and correlated it with patient prognosis. Functional assays, including cell proliferation, migration, and invasion tests, were conducted *in vitro*, and tumor growth was assessed *in vivo*. Additional mechanistic assays—including RNA-FISH, subcellular fractionation, and co-immunoprecipitation—were performed to determine the localization and molecular interactions of ZFHX4-AS1. The mechanistic interactions between ZFHX4-AS1, ZFHX4, SOX2, and the JAK-STAT pathway were investigated using gene expression analysis, protein-protein interaction studies, and signaling pathway activation assays.

**Results:**

Functionally, both ZFHX4-AS1 and ZFHX4 promote glioma cell proliferation, migration, and invasion in vitro and tumor growth *in vivo*. Mechanistically, ZFHX4-AS1 acts in cis to positively regulate the expression of ZFHX4. Crucially, we identified the stemness factor SOX2 as a key functional partner of ZFHX4. ZFHX4 and SOX2 physically interact and form a positive feedback loop, where each protein promotes the other’s expression. This regulatory circuit serves to amplify the oncogenic signal, robustly driving the malignant phenotype. Finally, we demonstrate that this signaling axis converges on the activation of the JAK-STAT pathway.

**Discussion:**

In conclusion, our study significantly expands upon the understanding of the ZFHX4-AS1 pathway in glioma. We demonstrate that ZFHX4-AS1 initiates an oncogenic signal which is powerfully amplified by a previously unidentified ZFHX4/SOX2 positive feedback loop. We further establish that this entire axis ultimately converges on the activation of the JAK-STAT pathway. This detailed ZFHX4-AS1/ZFHX4/SOX2/JAK-STAT axis represents a promising set of therapeutic targets for glioma treatment.

## 1 Introduction

Approximately 70% of primary intracranial tumors are gliomas, the most common and most aggressive type. Based on the classification of tumors in 2021 as published by the World Health Organization, there are four grades of gliomas: I, II, III, and IV, with each grade indicating the severity of the malignancy (1). Approximately 57% of all gliomas are glioblastomas, the most malignant histological type, with a high frequency of recurrence, and a low probability of cure for this type of cancer (2, 3). Although great progress has been made in multimodal combined therapy such as surgery, radiotherapy, chemotherapy, targeted therapy and supportive therapy in recent years, there is still a poor long-term prognosis and a very low long-term survival rate. As a consequence, finding sensitive biomarkers for glioma treatment is urgent.

RNAs that contain >200 nucleotides and have no or limited ability to code for proteins are known as lncRNAs (4). There are five categories that can be categorized based on the genomic location of lncRNAs in relation to neighboring genes: sense, antisense, bidirectional, intronic and intergenic (5–7). Based on where the cells are located, lncRNAs can be divided into nuclear and cytoplasmic types. Different locations of lncRNAs play different biological functions. Nuclear lncRNAs usually interfere with transcription and chromatin remodeling, while cytoplasmic lncRNAs can play a role by affecting RNA processing, mRNA stability and direct regulation of protein function (8, 9).

In recent years, researchers have become increasingly interested in identifying lncRNAs and understanding their functions in various aspects, such as trans-regulating distant genes and cis-regulating adjacent genes (10, 11). LncRNAs play a critical role in regulating a wide range of cancer-related cell functions, including those associated with gliomas (12–14). For instance, NEAT1 scaffolds EZH2, promoting glioblastoma cell growth and invasion (15). Furthermore, non-coding RNA HOTAIR can play a role in molecular signaling, modulating genes and their corresponding signal pathways (3, 16, 17). The use of lncRNAs as molecular biomarkers can also help predict the prognosis of patients (18–20).

ZFHX4-AS1 which was recently discovered is located on 8q21.13. There was research on the ceRNA network of bladder cancer which suggested that the expression of ZFHX4-AS1 was significantly higher in bladder cancers (21). Besides, a study on breast cancer found that ZFHX4-AS1 regulates the Hippo signaling pathway, which affects the progression of breast cancer (22). There is, however, no consensus regarding its role in gliomas.

To better understand the molecular mechanism of ZFHX4-AS1 and offer new perspectives on glioma treatment, this study investigates the role of ZFHX4-AS1 in glioma cell growth, proliferation, invasion, and metastasis. Our team’s preliminary work has previously reported a linear pathway where ZFHX4-AS1 upregulates its neighboring gene ZFHX4, which in turn promotes the expression of SOX2, thereby accelerating glioma progression (23). However, the precise regulatory interplay between ZFHX4 and SOX2, and the key downstream signaling pathways that are ultimately responsible for the observed malignant phenotypes, remain to be fully elucidated. Therefore, this study builds upon our initial findings to investigate the detailed molecular mechanisms, specifically focusing on a potential feedback loop and the ultimate convergence point of this oncogenic axis.

## 2 Materials and methods

### 2.1 Bioinformatics approaches

Data on pan-cancer was downloaded from the University of California, Santa Cruz Genome Browser (UCSC) database (https://xenabrowser.net/). Additionally, we performed log2 transformation on each expression value of ENSG00000253661 (ZFHX4-AS1) gene in each sample. Our analysis eliminated cancer species that had fewer than three samples within a single cancer species. Using R software (version 3.6.4), we calculated the expression differences between normal and tumor samples for 33 cancer species. Differential significance analysis was performed using unpaired Wilcoxon Rank Sum and Signed Rank Tests. In addition, we also obtained a set of high-quality The Cancer Genome Atlas(TCGA) prognostic data, which was previously published by the TCGA (24), and obtained TARGET followup data from UCSC’s Cancer browser (https://xenabrowser.net/datapages/) as a supplement and excluded samples with a follow-up time of less than 30 days. Additionally, log2 was used to modify each expression value. There was also an exclusion for cancer species with fewer than 10 samples in a single cancer species.

Our study examined the relationship between gene expression and prognosis in each tumor by using the Cox proportional hazards regression model developed using the coxph function of the R software package survival (version 3.2-7). A statistical test to determine prognostic significance was the logrank test.

The analysis of correlation between genes was performed at the online site GEPIA2 (http://gepia2.cancer-pku.cn/) and BioGRID (http://thebiogrid.org). For differential gene analysis on ZFHX4 and SOX2, we used RNAseq in level 3 HTSeq-Counts format from the TCGA database glioma project. The R package used was “DESeq2”. The screening criteria for identifying DEGs were an adjusted P-value (FDR) < 0.05 and an absolute log2 fold change (FC) > 1. We then performed Gene Ontology (GO), Kyoto Encyclopedia of Genes and Genomes (KEGG) enrichment analyses of the differential genes, mainly applying the clusterProfiler package (3.14.3). In addition to this, we also applied the clusterProfiler package (3.14.3) for gene set enrichment analysis(GSEA) of differential genes, with the gene set database being MSigDB Collections (https://www.gsea-msigdb.org/).

The analysis of correlation between genes was performed at the online site GEPIA2 (http://gepia2.cancer-pku.cn/) and BioGRID (http://thebiogrid.org). For differential gene analysis on ZFHX4 and SOX2, we used RNAseq in level 3 HTSeq-Counts format from the TCGA database glioma project. The R package used was “DESeq2”. The screening criteria for identifying DEGs were an adjusted P-value (FDR) < 0.05 and an absolute log2 fold change (FC) > 1. We then performed Gene Ontology (GO) and Kyoto Encyclopedia of Genes and Genomes (KEGG) enrichment analyses of the differential genes, mainly applying the clusterProfiler package (3.14.3). In addition to this, we also applied the clusterProfiler package (3.14.3) for gene set enrichment analysis (GSEA) of differential genes, with the gene set database being MSigDB Collections (https://www.gsea-msigdb.org/).

As part of ensuring data transparency and methodological consistency, the TCGA-LGG and TCGA-GBM datasets used in this study were retrieved from the UCSC Xena Browser, consistent with those analyzed in our 2023 preprint. In the present work, these datasets were reprocessed through an independent DESeq2 pipeline incorporating Benjamini–Hochberg FDR correction and extended for KEGG and GSEA analyses focusing on JAK–STAT–related signaling. Experimental data, including qRT-PCR, Western blot, and xenograft assays, were partly derived from the same experimental batches as those used in the preprint. These datasets were reanalyzed and extended to quantify transfection efficiency, evaluate JAK–STAT activation, and validate the newly identified ZFHX4/SOX2 positive feedback loop. No previously published figure or numerical result was reused verbatim; all analyses and visualizations were regenerated or reinterpreted within the expanded mechanistic framework. This transparent reuse and analytical extension ensure data integrity and reproducibility while providing mechanistic depth beyond the scope of the 2023 preprint.

### 2.2 Cell cultivation

For this study, we employed six glioma cell lines (U87, U138, U251, T98G, A172, and LN229) alongside normal human astrocytes (NHAs) as controls. All of these cell lines were provided by Procell Life Science. The cell lines U251 and U138 were cultured in RPMI1640 media (Gibco™,11875093). The cell lines A172 and LN229 were grown in DMEM (Gibco™, 11965092). The T98G and U87cell lines were maintained in MEM (Gibco™, 10370088), whereas the NHA cells were grown in astrocyte medium (AM, Sciencell™, #1801). Unless otherwise stated, all media were supplemented with 10% FBS (Gibco™, 12483020), 100 units of penicillin per ml, and 100 micrograms of streptomycin per ml (Pen-Strep Solution, Gibco™, 15070063). We cultivated all the cells at a humidity level of 5% in a humidified incubator at 37 °C.

### 2.3 Tissue samples

In this study, 30 glioma patients’ tumor specimens and tumor-adjacent non-tumor tissues were gathered from The Second Hospital of Hebei Medical University. The pathology department verified each sample. The clinical and pathological characteristics of the patient cohort, including the WHO grade distribution, are summarized in **Table 1**. These patients had never received any antineoplastic treatment. This study was conducted in accordance with the Declaration of Helsinki and was approved by the Institutional Ethics Committee of The Second Hospital of Hebei Medical University (Approval Code:2022-R005, Date of Approval: 2022-1-26). The requirement for individual written informed consent for this study was formally waived by the ethics committee because the research was conducted on fully anonymized, pre-existing pathological specimens, and it was deemed impracticable to re-contact the patients.

**Table 1:** The clinical and pathological characteristics of the patient cohort.

WHO grade	Case ID	Sex	Age (years)	Tumor location	Integrated diagnosis
Grade I (n = 5)	1	Female	12	Cerebellum	Pilocytic Astrocytoma
	2	Male	8	Optic Pathway	Pilocytic Astrocytoma
	3	Female	25	Frontal Lobe	Diffuse Astrocytoma
	4	Male	16	Temporal Lobe	Ganglioglioma
	5	Female	35	Parietal Lobe	Subependymal Giant Cell Astrocytoma
Grade II (n = 5)	6	Female	32	Frontal Lobe	Diffuse Astrocytoma
	7	Male	28	Frontal Lobe	Oligodendroglioma
	8	Female	45	Temporal Lobe	Diffuse Astrocytoma
	9	Male	38	Parietal Lobe	Oligodendroglioma
	10	Male	50	Frontal Lobe	Diffuse Astrocytoma
Grade III (n = 5)	11	Male	48	Frontal Lobe	Anaplastic Astrocytoma
	12	Female	41	Temporal Lobe	Anaplastic Oligodendroglioma
	13	Male	55	Parietal Lobe	Anaplastic Astrocytoma
	14	Female	60	Frontal Lobe	Anaplastic Astrocytoma
	15	Male	52	Occipital Lobe	Anaplastic Oligodendroglioma
Grade IV (n = 5)	16	Male	62	Frontal Lobe	Glioblastoma
	17	Female	58	Temporoparietal Lobe	Glioblastoma
	18	Male	68	Thalamus	Glioblastoma
	19	Female	45	Frontal Lobe	Glioblastoma
	20	Male	70	Cerebellum	Glioblastoma
Total (n = 20 cases)	–	10 M/10 F	Mean: 42.4	–	–

WHO, World Health Organization; M, male; F, female.

### 2.4 Cell transfection

By using small interfering RNAs (siRNAs) made by ZHONGSHITONTAU, ZFHX4-AS1, ZFHX4, and SOX2 in glioma cell lines were knocked down **Table 2**. A negative control (NC) was performed using nonspecific siRNAs. Lipofectamine 3000 (L3000015, Invitrogen) was used to transfect siRNAs at a final concentration of 60 pmol/106 cells. Afterward, 48 and 72 hours after transfection, respectively, RNAs and proteins were collected.

**Table 2:** List of siRNA sequences.

Target gene	Sense strand sequence(5’-3’)	Antisense sense strand sequence(5’-3’)
ZFHX4-AS1	GAGGUGUAAUGUUGUAUAAUG	UUAUACAACAUUACACCUCAG
ZFHX4	CGAUAGUAUUGGUAACAAAGA	UUUGUUACCAAUACUAUCGGU
SOX2	CCAAGACGCUCAUGAAGAAGG	UUCUUCAUGAGCGUCUUGGUU

The ZFHX4-AS1, ZFHX4 and SOX2 sequence was manufactured and cloned into the pcDNA 3.1 vector by Shanghai Genechen to create the ZFHX4-AS1, ZFHX4 and SOX2 overexpression plasmid (oe-ZFHX4-AS1, oe-ZFHX4, oe-SOX2). We used the blank pcDNA3.1 plasmid as a negative control plasmid (oe-NC). After 48 hours and 72 hours following transfection, RNAs and proteins were collected, respectively.

### 2.5 RT-PCR

TRIzolTM Plus RNA Purification Kit (Invitrogen, A33254) was utilized to extract RNA. cDNA was obtained by reverse transcription of the extracted RNA using PrimeScriptTM RT reagent (Takara, RR047A). In order to configure the reagents for qRT-PCR, TBGreen Premix ExTaq II (Takara, RR820A) instructions were followed and a quantitative PCR instrument was used to perform the analysis. An initial denaturation at 95 °C for 30 seconds, a denaturation at 95 °C for 5 seconds, an annealing at 60 °C for 34 seconds, and an extension period of 34 seconds were set as the parameters for the experiment. In order to calculate the relative expression, we used the 2−ΔΔCt method. The specific primer sequences are provided in **Table 3**.

**Table 3:** Primer sequences for qRT-PCR analysis.

Gene name	Primer direction	Sequence (5’-3’)
ZFHX4-AS1	Forward	GCCCCTGCCAACATCATTCAATTAC
	Reverse	ATCTGGGAAGCAAAGCATCTCTGTC
ZFHX4	Forward	CTTGCTTGAAGACCTAAAGCAG
	Reverse	GTGCTTGTTGCTGTAGTATCTG
SOX2	Forward	CAGCATGTCCTACTCGCAGCAG
	Reverse	CTGGAGTGGGAGGAAGAGGTAACC
GAPDH	Forward	GGAGCGAGATCCCTCCAAAAT
	Reverse	GGCTGTTGTCATACTTCTCATG
U6	Forward	GCTCGCTTCGGCAGCACATATAC
	Reverse	ATGATGGAACGCTTCACGAATTTGC

### 2.6 Western blot

The lysis buffer (Beyotime) was used to obtain proteins from cells and tissues, and then the BCA kit (Abcam, ab102536) was used to quantify the concentrations of proteins. After extraction or enrichment of the protein samples, 10% SDS-PAGE was used to separate the samples and polyvinylidene fluoride membranes were used to transfer them. The membranes were blocked for 30 mins. We incubated them overnight at 4 °C with primary antibodies against GAPDH (Abcam, ab8245, 1:10,000), ZFHX4 (Aviva Systems Biology, ARP39612_P050, 1:2,000), SOX2 (Abcam, ab171380, 1:1,500), JAK1 (Abcam, ab133666, 1:2,000), p-JAK1 (Abcam, ab138005, 1:2,500), STAT3 (Abcam, ab68153, 1:1,000), p-STAT3 (Abcam, ab267373, 1:800). After washing three times, the blots were incubated with the secondary antibody (Abcam; ab205718, 1:10,000). As a final step, enhanced chemiluminescence reagents (Beyotime, P0018AM) were used to develop protein signals.

### 2.7 Cell Counting Kit−8

Put 3×10^3^ cells per well in a 96-well plate. The viability of the cells should be evaluated at 1, 2, 3 and 4 days after transfection. For each well, place 10 μl of CCK-8 solution and incubate for two hours at 37 °C. Cell viability was evaluated at 450 nm.

### 2.8 Colony formation assay

A total of 600 transfected glioma cells were inoculated into 60mm cell culture dishes and cultured for 14 days in complete medium containing 10% FBS. Every 3 days, the medium should be changed. After 14 days, the cells were fixed with 4% paraformaldehyde, stained with Giemsa, and counted to determine the number of clones formed. The number of colonies was calculated by ImageJ.

### 2.9 EdU assay

5×10^3^ cells were inoculated with 100μl medium containing 10% FBS in 96-well plates until they attached. We labeled the cells with the Guangzhou Ruibo biological Cell-Light™Apollo488 Stain kit, added 100μl staining reaction solution, incubated at room temperature for two hours, and abandoned the staining reaction solution. It was washed three times with PBS containing 0.5% TritonX-100. Following the staining with Hoechst33342, the film was sealed and examined under a microscope.

### 2.10 Transwell assay

We starved transfected glioma cells in serum-free medium for 24 hours before inoculating them in chambers on Transwells (8 mm; BD Biosciences) coated with 40 μl Matrigel or left uncoated with Matrigel. Each chamber was populated with 1×10^5^cells.Complete medium containing 10% FBS was added to the lower chamber. 24 hours later, the cells in the upper chamber were wiped off, fixed in 4% paraformaldehyde and counterstained with 0.1% crystal violet.

### 2.11 Wound healing

The transfected U251 cells (1×10^5^) were inoculated into 6-well plates. Upon reaching 100% confluence, the cells were scratched with a 10 μL pipette tip. We gently washed away the detached cells with PBS. The scratching of cells was assessed with a microscope at 0 hour and 24 hour in the same field of view. The width of the scratches was analyzed using ImageJ.

### 2.12 Xenograft in nude mice model

For the subcutaneous injection of tumor cells, mice were anesthetized using an intraperitoneal injection of pentobarbital sodium at a dose of 50 mg/kg. Transfected U251 cells (1​x 106​) were injected subcutaneously into thymus-free BALB/c nude mice (4 weeks, male). For the *in vivo* studies, a total of 20 mice were used. To investigate the function of ZFHX4-AS1, 10 mice were randomly divided into an oe-NC group and an oe-ZFHX4-AS1 group (n=5 per group). To investigate the function of ZFHX4, another 10 mice were randomly divided into an oe-NC group and an oe-ZFHX4 group (n=5 per group). Animals that have been treated were housed in SPF-class housing of laboratory. The volume of the subcutaneous graft tumor was measured every 7 days, and the volume was calculated using the formula V=(length×width2)/2. After 28 days, the animals were euthanized by cervical dislocation before the tumor tissues were collected, weighed, and photographed. All animal experiments were performed in accordance with institutional guidelines and were formally approved by the Institutional Animal Care and Use Committee of The Second Hospital of Hebei Medical University (Approval Code: 2022-AE005, Date of Approval: 2022-1-26).

The animal experiments were conducted in accordance with ethical standards and approved by the Institutional Animal Care and Use Committee. Each group initially included ten mice, and data from five mice per group (n = 5) were used for analysis after excluding non-viable cases, following the principle of using the minimum number of animals required for scientific validity. Tumor formation and growth were assessed through direct observation and measurement of tumor volume and weight. Histological analyses (e.g., H&E or Ki67 staining) were not performed, as macroscopic tumor assessment was considered sufficient to meet the experimental aims while minimizing animal use.

### 2.13 Fluorescence *in situ* hybridization

ZFHX4-AS1 FISH probes are manufactured by RiboBio (Guangzhou, China). In confocal dishes, U251 cells were seeded 24 hours before hybridization. Afterwards, cells were fixed, prehybridized, and hybridized with FISH probes using a hybridization buffer. The slides were cleaned, dehydrated, and stained with DAPI. Immunofluorescence images were captured using an Olympus microscope (Tokyo, Japan).

### 2.14 Subcellular fractionation assay

Cytoplasmic and nuclear fractions were isolated from glioma cells using the PARIS kit (Invitrogen, AM1921). The relative expression of ZFHX4-AS1, GAPDH and U6 was subsequently examined using RT-qPCR to determine the expression of ZFHX4-AS1 in the nucleus as well as in the cytoplasm.

### 2.15 Co-immunoprecipitation assay

Proteins were extracted using RIPA Lysis Buffer. A small fraction of the lysate was reserved as the ‘Input’ control for subsequent Western blot analysis. The remaining lysate was incubated with an anti-SOX2 antibody (Abcam, ab171380) overnight at 4 °C with gentle rotation. Subsequently, pre-treated protein A/G-agarose beads were added to the mixture and incubated for an additional 2 hours at 4 °C to capture the immune complexes. The beads were then collected by centrifugation and washed three times with lysis buffer to remove non-specifically bound proteins. Finally, the immunoprecipitated proteins were eluted from the beads by boiling in SDS loading buffer for 10 minutes, and the samples were analyzed by Western Blotting.

### 2.16 Statistical analyses

Our analysis and processing of the data were conducted with GraphPad Prism 8.0, which employed a t-test to determine the variance between two groups and a one-way ANOVA to determine the variance between multiple groups. The mean value (SD) of each experiment was calculated from three repetitions. Kaplan-Meier curves were used to determine the prognosis of patients. The Log-rank test was used to assess factors that were associated with overall survival. Differences between groups that *P* < 0.05 were considered statistically significant.

## 3 Results

### 3.1 Expression and prognosis analysis of ZFHX4-AS1 in pan−cancer

**Figure 1:**
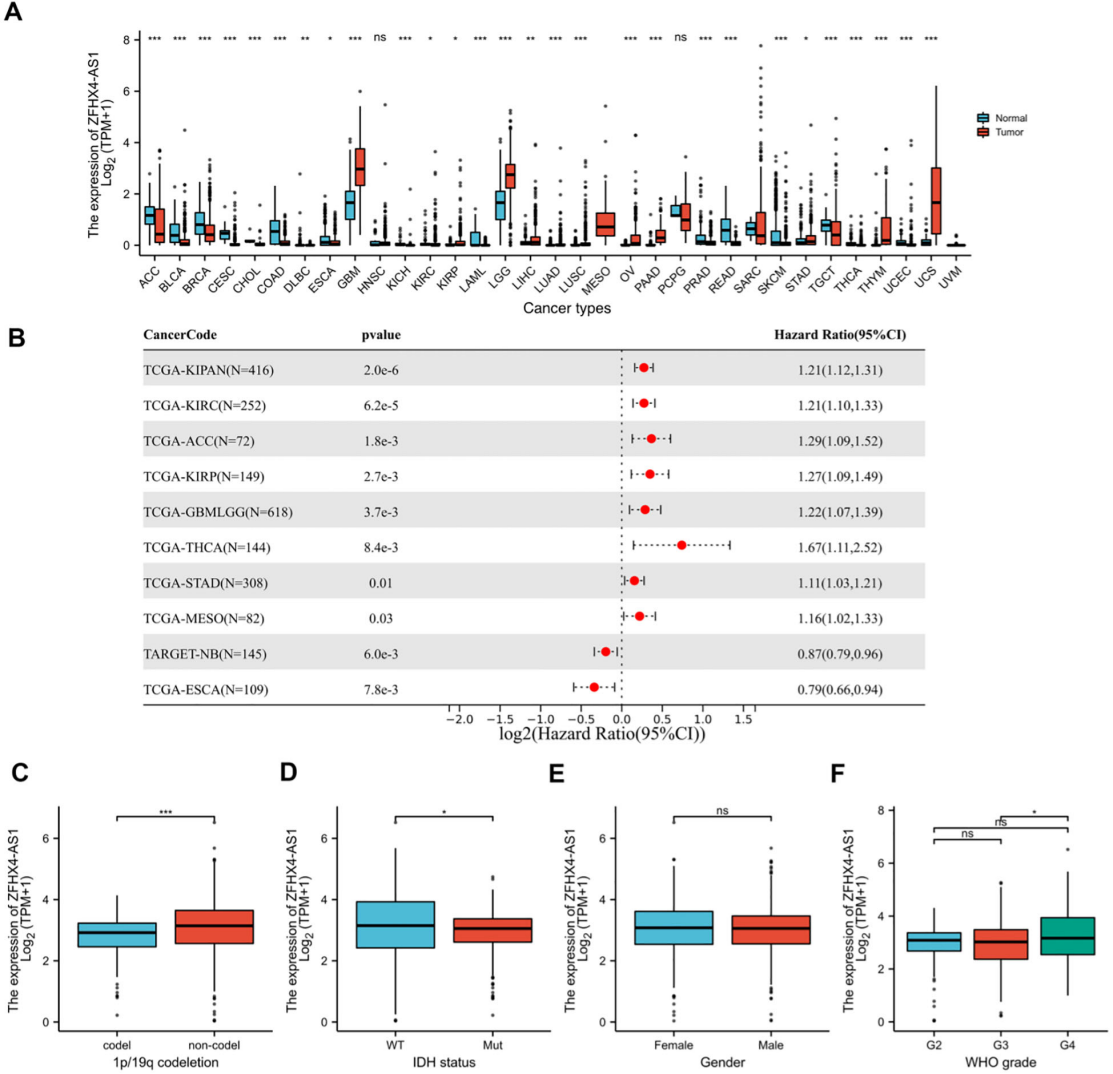
Bioinformatics analysis of ZFHX4-AS1. **(A)** Expression of ZFHX4-AS1 in pan-cancer data of TCGA. **(B)** Prognostic analysis of ZFHX4-AS1 in pan-cancer. **(C)** ZFHX4-AS1 expression is higher in gliomas of 1p19q non-codel type. **(D)** IDH-wildtype gliomas express a higher level of ZFHX4-AS1. **(E)** The expression of ZFHX4-AS1 was not significantly different between the gender. **(F)** The expression level of ZFHX4-AS1 was significantly higher in WHO IV gliomas.(ns no significance, *P < 0.05, **P < 0.01, ***P < 0.001).

In comparing data from 33 tumor species, we observed significant up-regulation of ZFHX4-AS1 in 11 tumors using R software, such as GBMLGG, LUAD, STAD, LUSC, LIHC, OV, PAAD, UCS, KIRP, THYM. We observed significant down-regulation in 17 kinds of tumors, such as BRCA, CESC, ESCA, COAD, PRAD, KIRC, SKCM, BLCA, THCA, READ, TGCT, ACC, KICH, CHOL, DLBC, LAML, UCEC (**Figure 1A**).

According to our analysis of the relationship between ZFHX4-AS1 expression and tumor prognosis, 11 tumor types with high expression of ZFHX4-AS1 had poor prognosis, whereas 2 tumor types with low expression had poor prognosis (**Figure 1B**).

In order to clarify the role of ZFHX4-AS1 in the occurrence and development of gliomas, we analyzed the relationship between ZFHX4-AS1 and clinical characteristics in the TCGA database, including gender, WHO grade, 1p19q deletion, and status of IDH. It was shown that ZFHX4-AS1 expression levels are also different when 1p19q deletions (**Figure 1C**) and IDH mutations differ (**Figure 1D**). Gender did not show a statistically significant difference (**Figure 1E**). However, ZFHX4-AS1 expression was significantly correlated with tumor grade, showing a markedly higher level in WHO Grade IV gliomas compared to lower-grade tumors (**Figure 1F**).

### 3.2 ZFHX4-AS1 is highly expressed in tissues and cells

**Figure 2:**
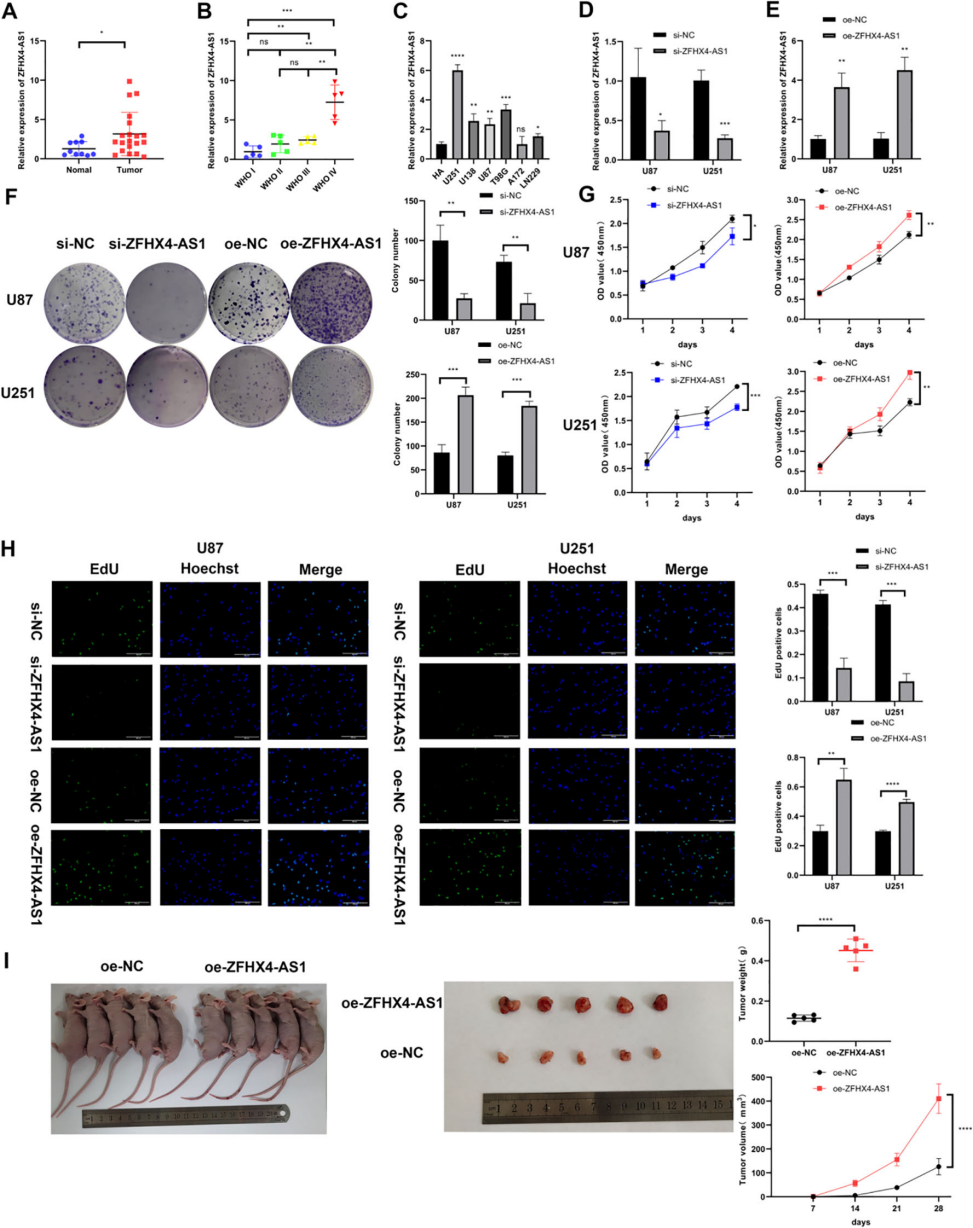
ZFHX4-AS1 is highly expressed in glioma and promotes glioma cell proliferation *in vitro*/vivo. **(A-C)** RT-PCR analysis of ZFHX4-AS1 expression in glioma tissues and cells. **(D, E)** RT-PCR analysis of ZFHX4-AS1 overexpression and knockdown efficiency. **(F)** Colony formation assay for assessing proliferation of transfected glioma cells. **(G)** CCK-8 assay detecting the effect of ZFHX4-AS1 on viability of transfected glioma cells. **(H)** EdU assay examining the effect of ZFHX4-AS1 on proliferation of transfected glioma cells. **(I)** Tumor weight/volume measurements in nude mice every 7 days to assess *in vivo* growth rate (n = 5). All results shown as mean ± SD; analyzed by unpaired t-tests (ns: no significance, *P < 0.05, **P < 0.01, ***P < 0.001, ****P < 0.0001).

To experimentally validate our bioinformatic findings, we performed RT-PCR analysis on our collected patient cohort. This analysis revealed that ZFHX4-AS1 expression was significantly elevated in glioma tissues compared to their matched tumor-adjacent non-tumor tissues (**Figure 2A**). Furthermore, this upregulation was most pronounced in WHO Grade IV gliomas, which showed significantly higher expression than lower-grade tumors (**Figure 2B**). To confirm this trend using a well-defined *in vitro* model, we compared ZFHX4-AS1 levels across six glioma cell lines and the NHA control. The results have shown that glioma cell lines, except for A172, expressed significantly higher levels of ZFHX4-AS1 compared to NHA cells (**Figure 2C**).

### 3.3 ZFHX4-AS1 promotes glioma cell proliferation *in vitro*

To investigate the function of ZFHX4-AS1 in glioma, we modulated its expression using siRNAs for suppression and plasmids for overexpression. The efficacy of knockdown and overexpression was confirmed by RT-PCR. In U87 and U251 cells, si-ZFHX4-AS1 and oe-ZFHX4-AS1 respectively reduced the mRNA expression of ZFHX4-AS1 by more than 65% and overexpressed it by more than 3.5 times. (**Figures 2D,E**). Subsequent functional assays, including colony formation, CCK-8, and EdU assays, consistently demonstrated that silencing ZFHX4-AS1 inhibited glioma cell proliferation, whereas its overexpression significantly promoted proliferation (**Figures 2F–H**).

### 3.4 ZFHX4-AS1 promotes the growth of glioma cells *in vivo*

U251 cells that were transfected with oe-ZFHX4-AS1 or oe-NC were stereotactically implanted in nude mice. We measured tumor volume every seven days, and extracted tumor tissue after 28 days for weighing. The results showed that the tumor growth rate was significantly stimulated by overexpression of ZFHX4-AS1 (**Figure 2I**). It was found that the oe-ZFHX4-AS1 group had 410.54 mm^3^ volume compared to 125.99mm^3^ volume in the control group, while the oe-ZFHX4-AS1 group had 451.52 mg mass compared to 114.72mg mass in the control group. According to the experimental results, overexpression of ZFHX4-AS1 stimulates tumor growth to a significant degree.

### 3.5 ZFHX4-AS1 promotes glioma cell invasion and migration

**Figure 3:**
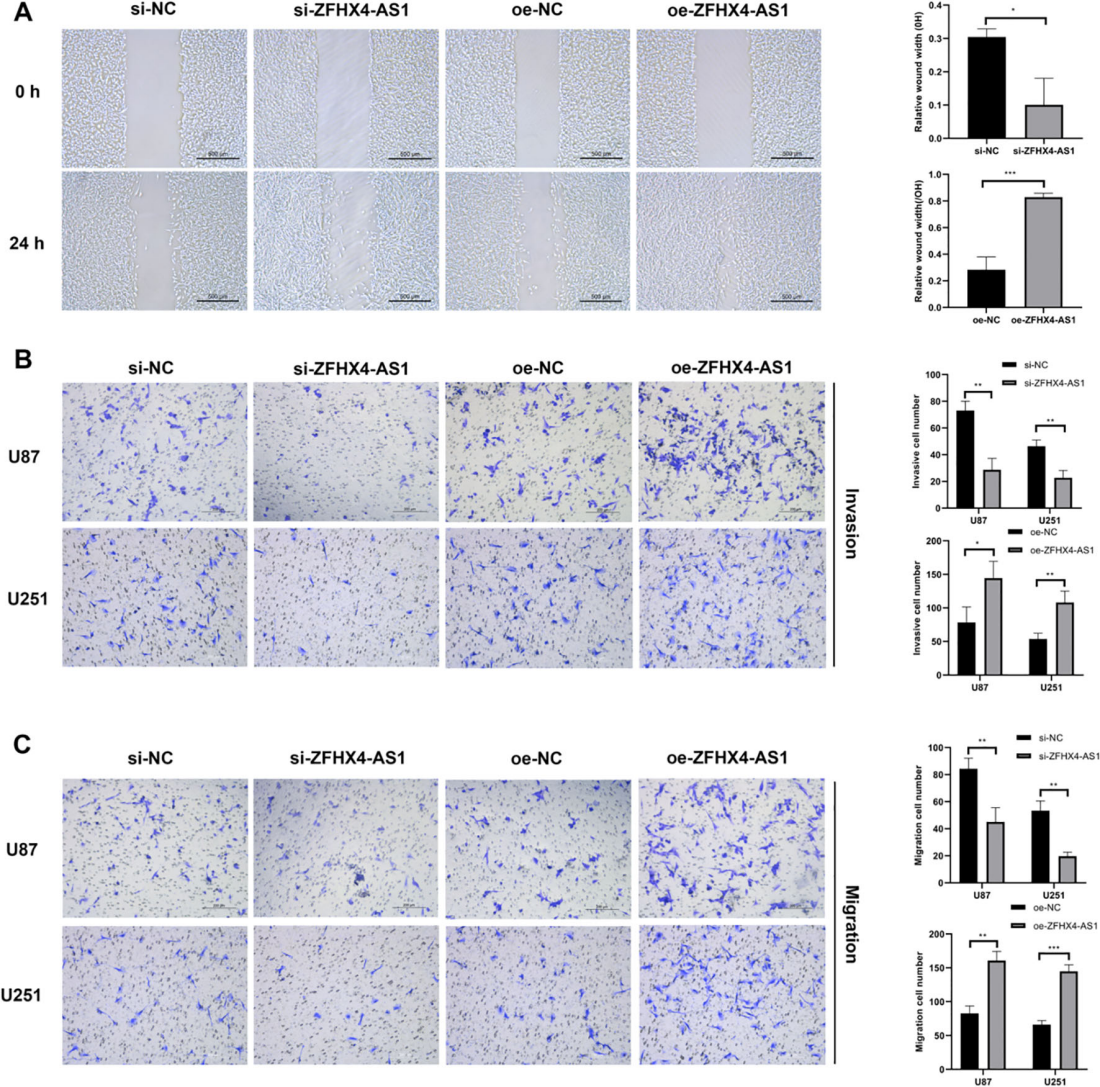
ZFHX4-AS1 promotes glioma cell invasion and migration. **(A)** Wound healing assay demonstrated that ZFHX4-AS1 overexpression enhanced U251 cell migration, while knockdown inhibited it. **(B, C)** Transwell assays revealed that ZFHX4-AS1 overexpression increased invasion and migration of U87 and U251 cells, whereas knockdown significantly reduced these abilities. All results shown as mean ± SD from three independent experiments; analyzed by unpaired t-tests (*P < 0.05, **P < 0.01, ***P < 0.001).

The role of ZFHX4-AS1 in glioma cell invasion and migration was investigated using wound healing and Transwell assays. Knockdown of ZFHX4-AS1 significantly inhibited the migration and invasion of glioma cells. Conversely, overexpression of ZFHX4-AS1 significantly enhanced these abilities, indicating that ZFHX4-AS1 promotes the invasion and migration of glioma cells (**Figures 3A–C**).

### 3.6 Glioma cells express ZFHX4-AS1 in both their nucleus and cytoplasm

**Figure 4:**
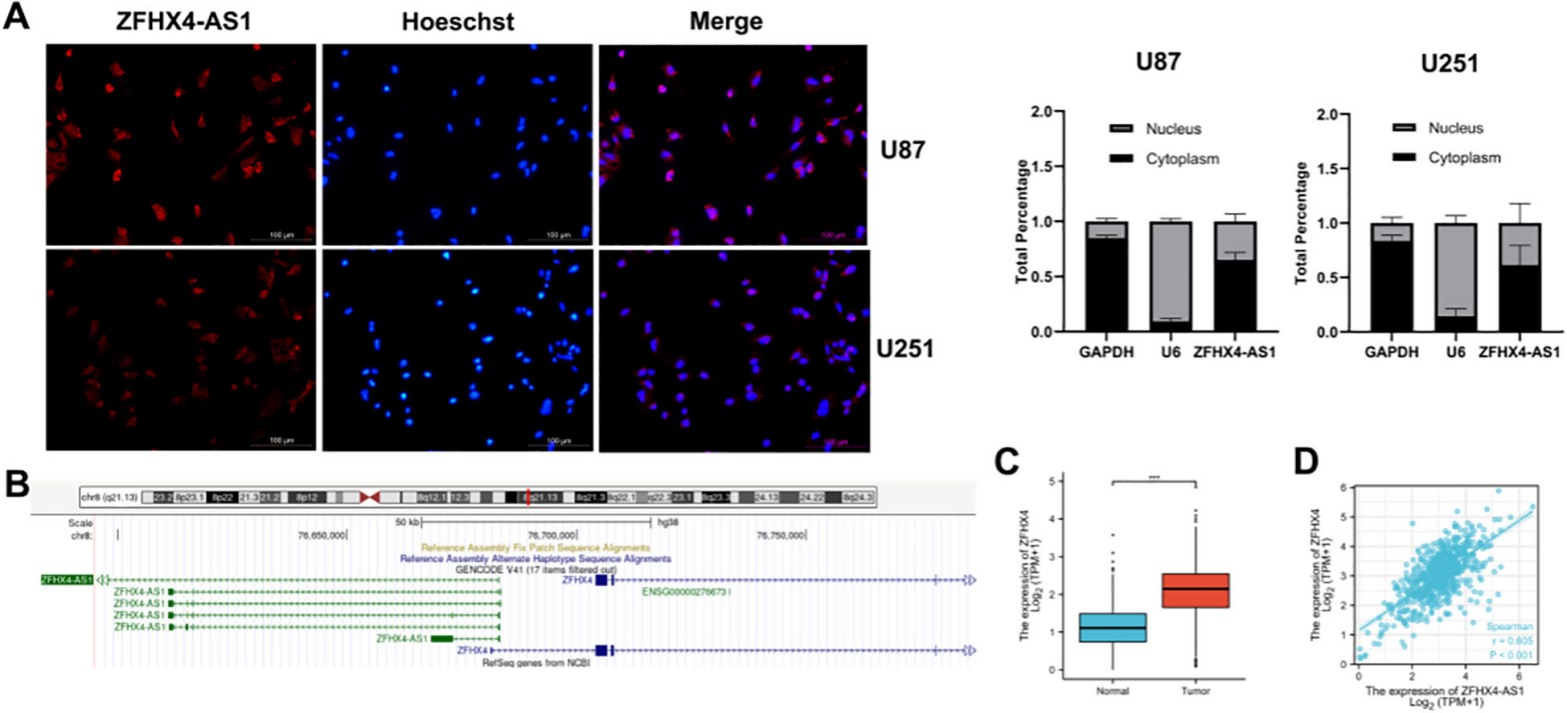
Subcellular localization of ZFHX4-AS1 and its adjacent gene. **(A)** RNA-FISH and subcellular fractionation assays determined the localization of ZFHX4-AS1. **(B)** UCSC database analysis identified ZFHX4 as an adjacent gene to ZFHX4-AS1. **(C)** Integrated TCGA and GTEx data revealed significantly elevated ZFHX4 expression in glioma tissues (***P < 0.001). **(D)** ZFHX4 expression positively correlated with ZFHX4-AS1 in TCGA and GTEx datasets.

In this study, we used FISH and subcellular fractionation assays to determine the subcellular localization of ZFHX4-AS1 with the aim of exploring its mechanism of effect on malignant biological behavior. According to the results, both the nuclear and the cytoplasms were found to express ZFHX4-AS1 (**Figure 4A**).

### 3.7 The nearby gene ZFHX4 is highly expressed in gliomas

**Figure 5:**
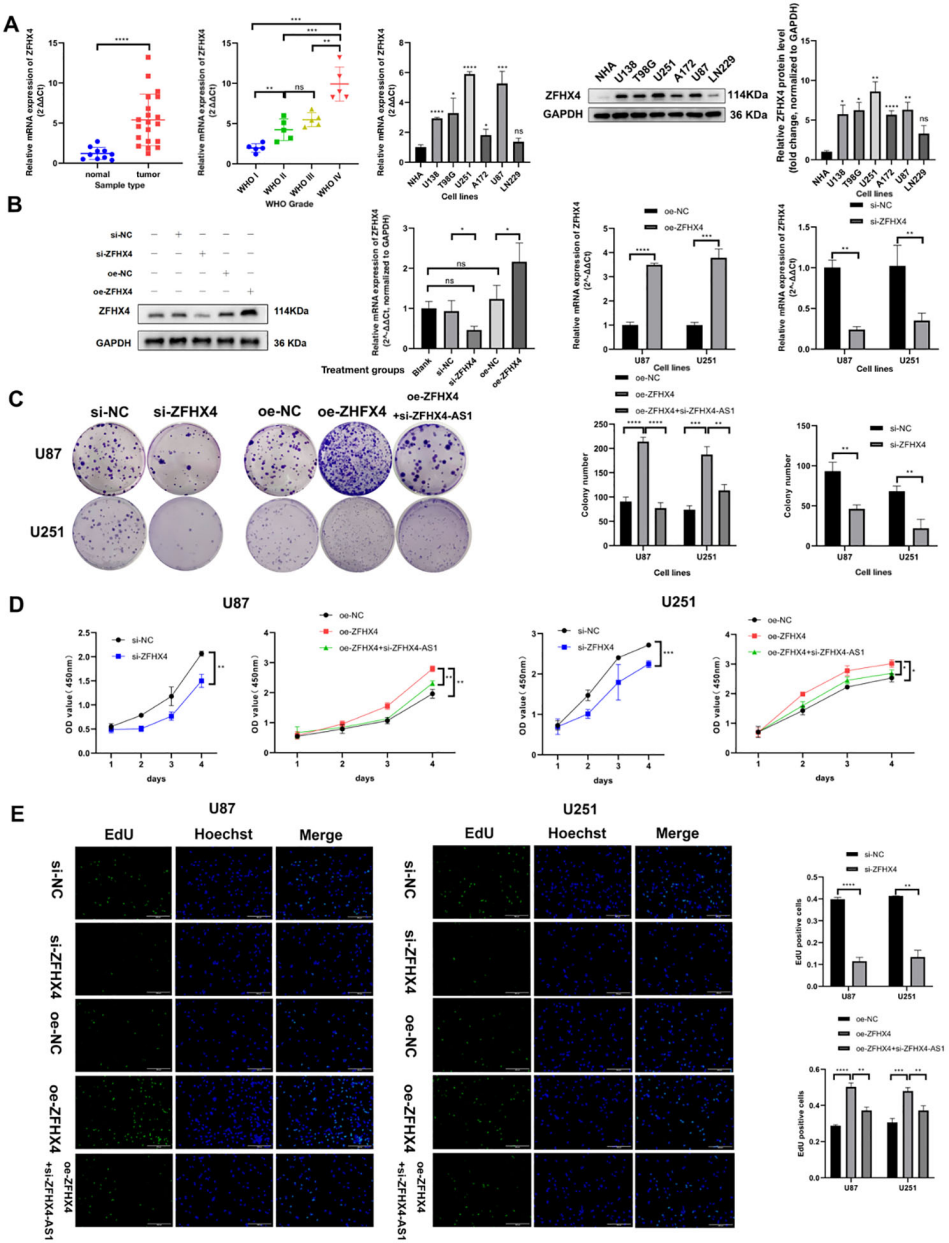
ZFHX4 is highly expressed in glioma and promotes glioma cell proliferation. **(A)** RT-qPCR and Western blotting confirmed elevated ZFHX4 expression in glioma tissues and cell lines. **(B)** RT-qPCR and Western blotting validated ZFHX4 overexpression and knockdown efficiency. **(C)** Colony formation assay assessed proliferation in transfected glioma cells. **(D)** CCK-8 assay evaluated the impact of ZFHX4 on cell viability. **(E)** EdU assay examined ZFHX4’s effect on proliferation. All results expressed as mean ± SD (triplicate experiments); analyzed by unpaired t-tests (ns: no significance, *P < 0.05, **P < 0.01, ***P < 0.001, ****P < 0.0001).

Using the UCSC database, we were able to determine that ZFHX4 is a nearby gene to ZFHX4-AS1 (**Figure 4B**). As a result of the data from the TCGA and GTEx databases, ZFHX4 expression exhibited a significant increase in glioma tissues (**Figure 4C**), and ZFHX4 expression was positively correlated with ZFHX4-AS1 (**Figure 4D**). To experimentally validate our bioinformatic findings, we performed RT-PCR analysis on our collected patient cohort. This analysis revealed significantly elevated ZFHX4 expression in glioma tissues compared to matched tumor-adjacent non-tumor tissues. Furthermore, this upregulation was most pronounced in WHO grade IV gliomas, where expression was significantly higher than in lower-grade tumors. To confirm this trend using well-defined *in vitro* models, we compared ZFHX4 levels via RT-PCR and Western Blotting across six glioma cell lines and NHA controls. The results demonstrated that glioma cell lines, except for Ln229, expressed significantly higher levels of ZFHX4 compared to NHA cells (**Figure 5A**).

### 3.8 ZFHX4 promotes the proliferation, invasion and migration in glioma

**Figure 6:**
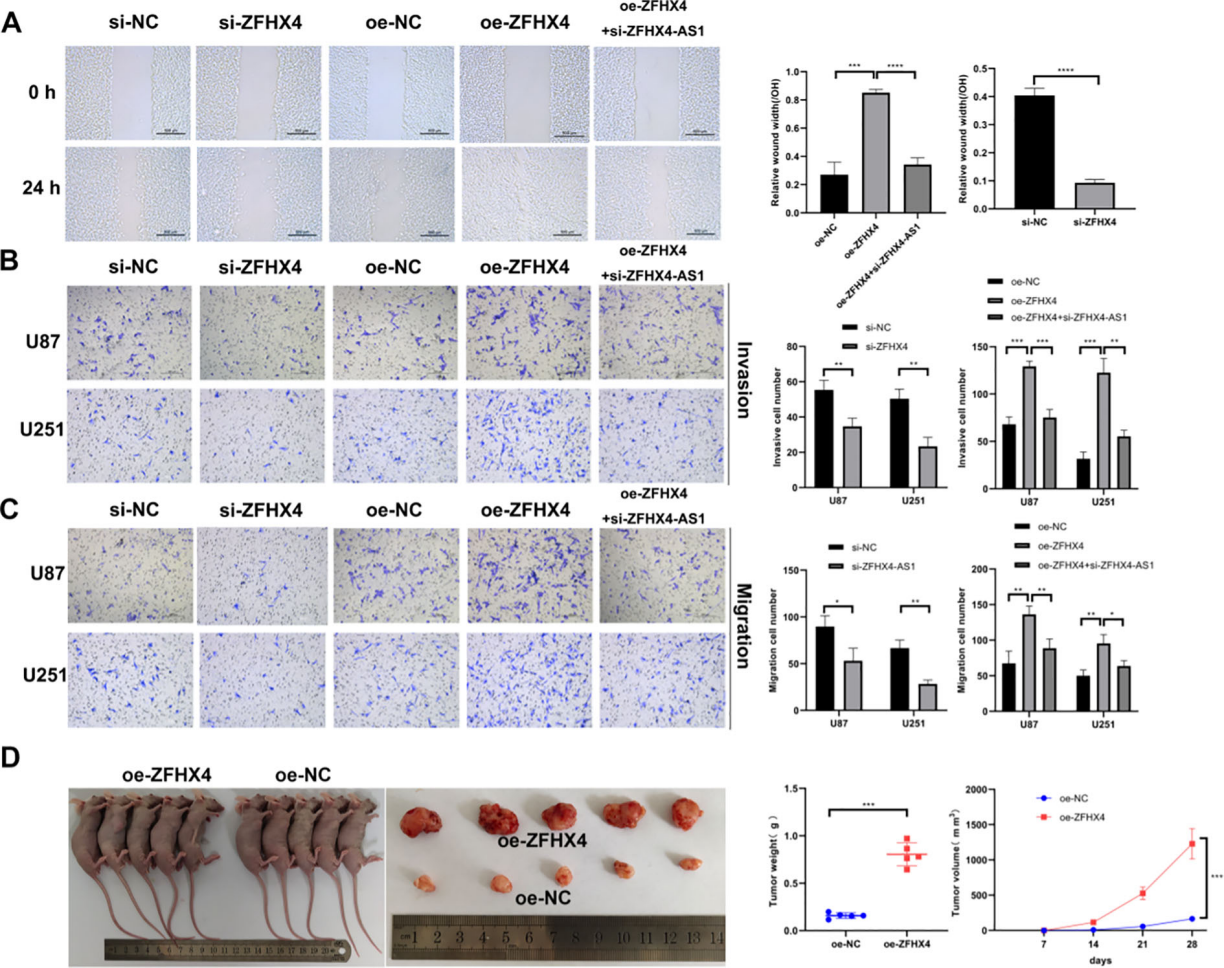
ZFHX4 promotes glioma cell invasion, migration and increases glioma growth rate. **(A)** Wound healing assays demonstrated that: ZFHX4 overexpression enhanced U251 cell migration, while knockdown inhibited it; Co-transfection with ZFHX4-AS1 siRNA attenuated the pro-migratory effect of ZFHX4 overexpression. **(B-C)** Transwell assays revealed: ZFHX4 overexpression increased invasion/migration in U87 and U251 cells;ZFHX4-AS1 knockdown reversed the enhanced invasion/migration induced by ZFHX4 overexpression. **(D)** Tumor volume/weight measurements in nude mice showed ZFHX4-dependent acceleration of *in vivo* glioma growth (n=5). All data expressed as mean ± SD; analyzed by unpaired t-tests (*P < 0.05, **P < 0.01, ***P < 0.001, ****P < 0.0001).

We used siRNA to knock down ZFHX4 expression and overexpressed it by plasmid to investigate its role in glioma. RT-PCR and Western Blot assays were performed to confirm ZFHX4 was successfully silenced and overexpressed in glioma cell lines. In U87 and U251 cells, si-ZFHX4 and oe-ZFHX4 respectively reduced the mRNA expression of ZFHX4 by more than 65% and overexpressed it by more than 3 times. (**Figure 5B**). Based on colony formation assay (**Figure 5C**), CCK-8 assay (**Figure 5D**) and EdU assay (**Figure 5E**), it was found that silencing ZFHX4 inhibited glioma cell proliferation, while enhanced ZFHX4 expression increased cell proliferation. In addition, we conducted healing assay (**Figure 6A**),cell invasion (**Figure 6B**) and migration (**Figure 6C**) experiments and found that knocking down of ZFHX4 significantly decreased the migration and invasion abilities of glioma cells as compared to control cells. Meanwhile, it was found that overexpression of ZFHX4 significantly enhanced the migratory and invasive abilities of cells.

### 3.9 ZFHX4 promotes the growth of glioma cells *in vivo*

The tumourigenicity of ZFHX4 was investigated in our experiments as well. A significant increase in tumor growth rate was observed after ZFHX4 was overexpressed on days 7, 14, 21 and 28 after implantation. The final volume of the oe-ZFHX4 group was 1228.85 mm^3^ compared to 166.3 mm^3^ in the control group, and the mass of the oe-ZFHX4 group was 804.62 mg compared to 157.9 mg in the control group. These results demonstrate that ZFHX4 stimulates the proliferation of glioma cells *in vivo* (**Figure 6D**).

### 3.10 ZFHX4-AS1 modulates proliferation, invasion and migration of glioma cells via regulating its nearby gene ZFHX4

**Figure 7:**
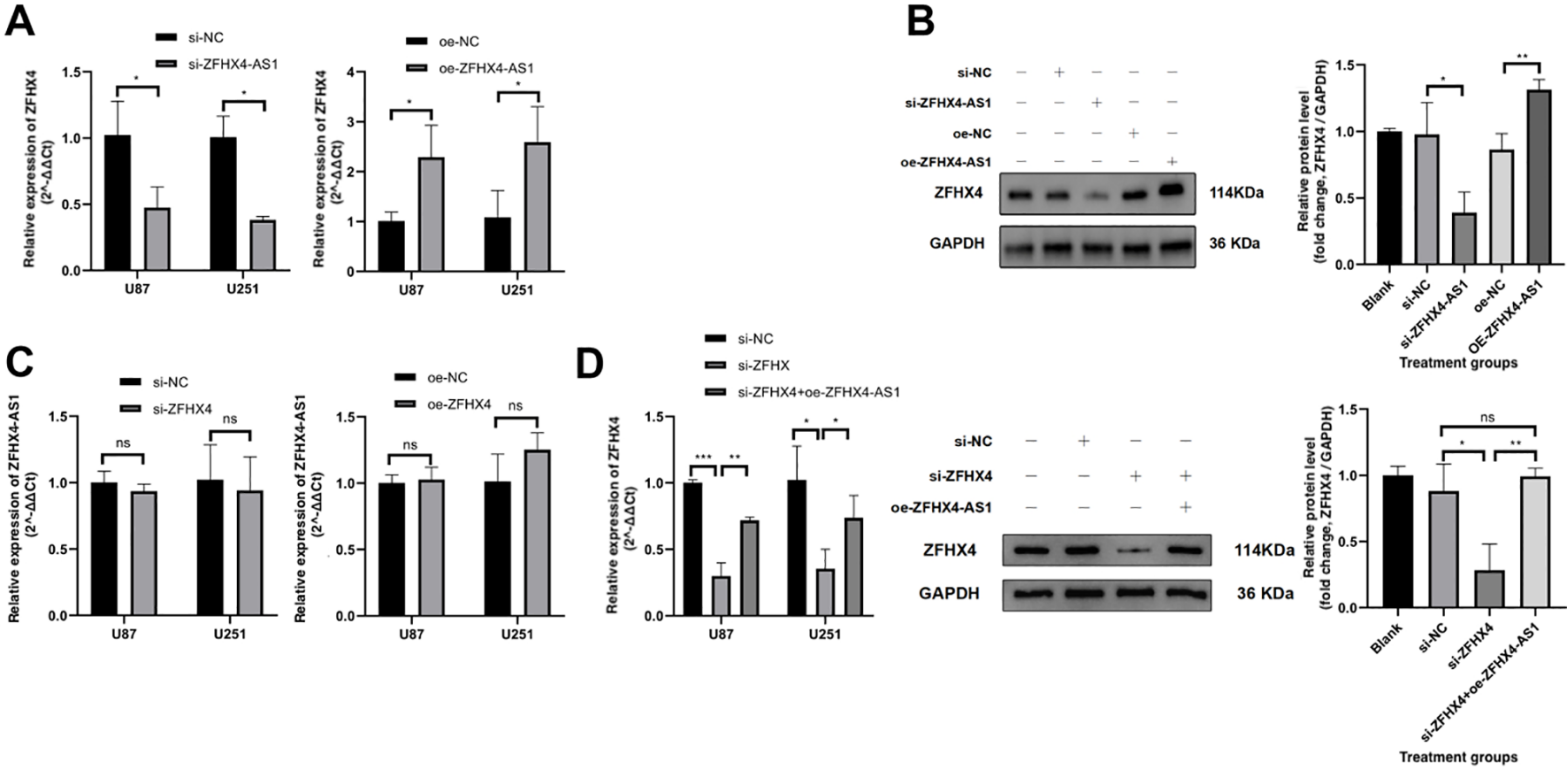
Regulatory relationship between ZFHX4-AS1 and ZFHX4. **(A-B)** RT-qPCR and Western blotting demonstrated that ZFHX4-AS1 knockdown/overexpression significantly altered ZFHX4 expression. **(C)** RT-qPCR confirmed no detectable changes in ZFHX4-AS1 expression following ZFHX4 perturbation (ns). **(D)** Co-transfection assays showed ZFHX4-AS1 overexpression rescued ZFHX4 expression suppressed by siRNA. All data represent mean ± SD (triplicates); analyzed by unpaired t-tests (ns: no significance, *P < 0.05, **P < 0.01, ***P < 0.001, ****P < 0.0001).

Following the knockdown of ZFHX4-AS1 and the overexpression of ZFHX4-AS1, there were substantial changes in the expression of ZFHX4 in glioma cells as determined by RT-PCR (**Figure 7A**) and Western Blot (**Figure 7B**) analysis. The results demonstrated that ZFHX4 expression decreased and increased in glioma cells following knockdown and overexpression of ZFHX4-AS1. We also detected ZFHX4-AS1 expression in glioma cells that overexpressed and knocked down ZFHX4. The expression of ZFHX4-AS1 did not change with the change in ZFHX4, which suggests that ZFHX4-AS1 and ZFHX4 may have a one-way regulatory relationship (**Figure 7C**). In addition, silencing ZFHX4 followed by overexpression of ZFHX4-AS1 rescued ZFHX4 expression in glioma cells (**Figure 7D**). Furthermore, the enhanced proliferation, migration, and invasive ability induced by ZFHX4 overexpression were significantly reversed by the simultaneous knockdown of ZFHX4-AS1 (**Figures 5C–E**, **Figure 6A–C**).The results of these studies confirmed that ZFHX4-AS1 modulates the proliferation, invasion, and migration of glioma cells through its nearby gene ZFHX4.

### 3.11 Analysis of differentially expressed genes

**Figure 8:**
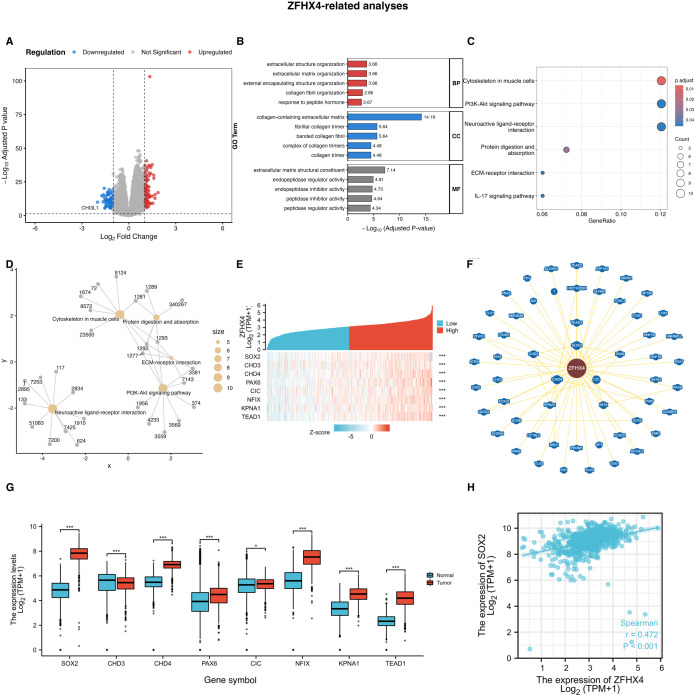
Functional analysis of ZFHX4 and identification of its key interacting partner SOX2 (updated with FDR correction). **(A)** Volcano plot showing differentially expressed genes (DEGs) between low- and high-ZFHX4 expression groups in the TCGA cohort. Red dots represent up-regulated genes, and blue dots represent down-regulated genes. **(B)** Bar plot of Gene Ontology (GO) enrichment analysis for the DEGs, displaying top terms in Biological Process (BP), Cellular Component (CC), and Molecular Function (MF). **(C)** Bubble plot of Kyoto Encyclopedia of Genes and Genomes **(KEGG)** pathway enrichment analysis. Bubble size corresponds to the number of enriched genes, and the color scale represents the adjusted P-value. **(D)** Gene-concept network illustrating the relationships between key DEGs and the most significant KEGG pathways. **(E)** Heatmap of ZFHX4-correlated genes. **(F)** Protein interaction network of ZFHX4-associated genes. **(G)** Relative expression of ZFHX4-network genes in glioma vs. normal tissues (TCGA). **(H)** Correlation between ZFHX4 and SOX2 expression (TCGA; ***P < 0.001).

We investigated the differentially expressed genes (DEGs) among the highly and lowly ZFHX4 expressed groups in TCGA to determine the mechanisms by which ZFHX4 regulates glioma development and occurrence. Using the screening criteria of an adjusted P-value (FDR) < 0.05 and an absolute log2 fold change > 1, we identified 105 down-regulated and 89 up-regulated genes (**Figure 8A**). To understand the biological functions associated with these differentially expressed genes (DEGs), we conducted Gene Ontology (GO) and Kyoto Encyclopedia of Genes and Genomes (KEGG) pathway enrichment analyses. The GO analysis revealed that the DEGs were significantly enriched in biological processes related to the tumor microenvironment and cellular structure, such as extracellular structure organization and response to peptide hormone. Key cellular component terms included collagen-containing extracellular matrix, and prominent molecular functions were associated with extracellular matrix structural constituent and peptidase regulator activity (**Figure 8B**). This suggests that ZFHX4 may extensively remodel the extracellular matrix to facilitate tumor invasion and migration. Furthermore, KEGG pathway analysis highlighted several well-established cancer-related signaling pathways. The DEGs were significantly enriched in the PI3K-Akt signaling pathway, ECM-receptor interaction, and the IL-17 signaling pathway (**Figure 8C**), all of which are known to be crucial for cell proliferation, survival, and inflammation in cancer. A gene-concept network was constructed to visualize the intricate connections between the key DEGs and these enriched KEGG pathways (**Figure 8D**). This integrated analysis provides a broad functional landscape, strongly indicating that ZFHX4 orchestrates a wide range of pro-tumoral activities, setting the stage for the investigation of its key molecular partners.

### 3.12 ZFHX4 binds to SOX2 and promotes its expression, thereby contributing to the malignant biological behavior of gliomas

We analyzed the proteins interacting with ZFHX4 by BioGRID. Eight key proteins with strong interactions were identified, including SOX2, CHD3, CHD4, PAX6, CIC, NFIX, KPNA1 and TEAD1, which were positively correlated with the expression of ZFHX4 (**Figures 8E, F**). Upon examining the relative expression of these eight genes, it was discovered that they were all highly expressed in gliomas (**Figure 8G**). As a follow-up study, we selected SOX2, which had the highest relative expression and differential expression. Furthermore, the expression of SOX2 showed a strong positive correlation with ZFHX4 in glioma (**Figure 8H**).

**Figure 9:**
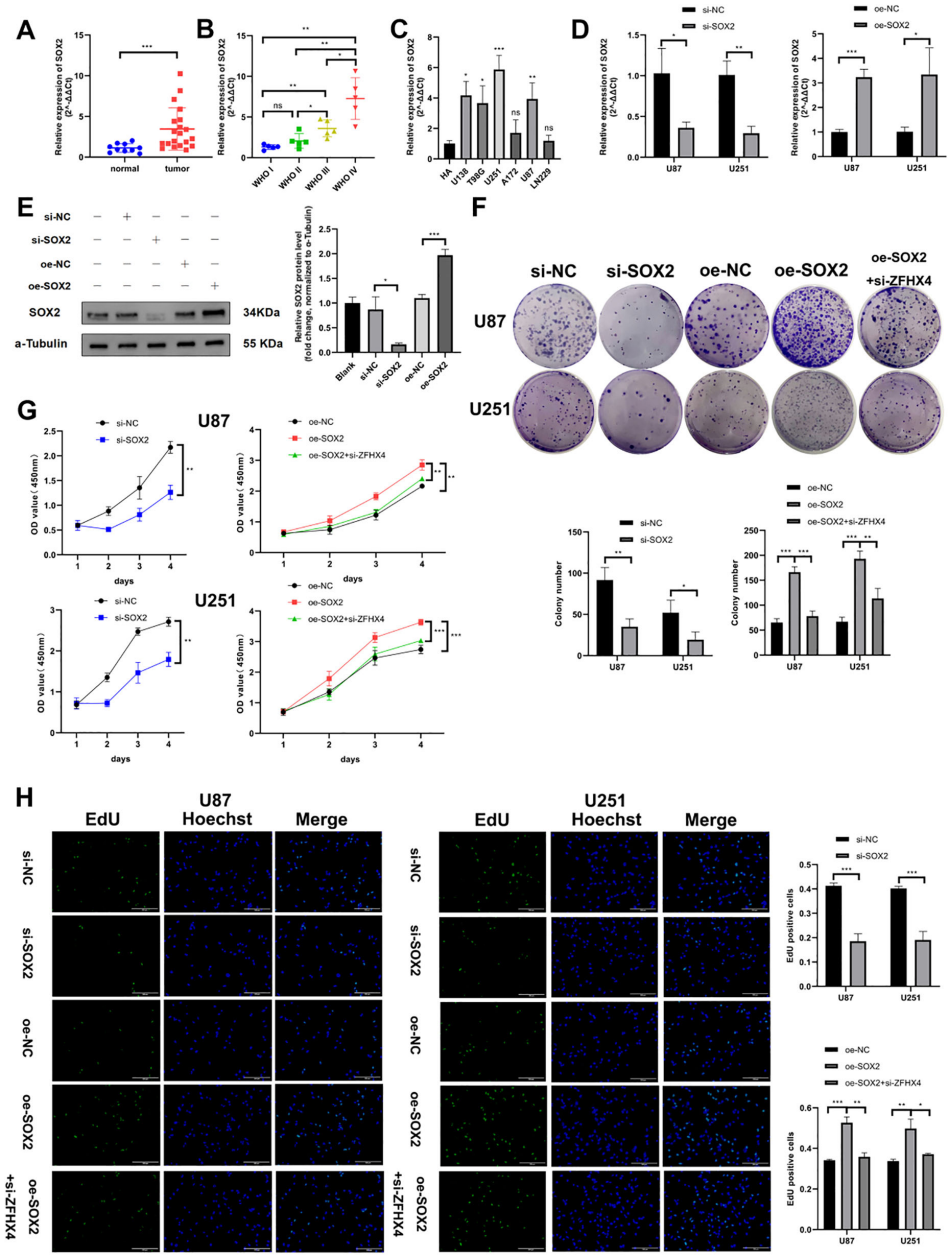
SOX2 is highly expressed in glioma and promotes glioma cell proliferation. **(A-C)** RT-qPCR analysis confirmed elevated SOX2 expression in glioma tissues and cell lines. **(D-E)** RT-qPCR and Western blotting validated SOX2 overexpression and knockdown efficiency. **(F)** Colony formation assay demonstrated altered proliferative capacity in SOX2-modulated cells. **(G)** CCK-8 assay revealed SOX2-dependent changes in cell viability. **(H)** EdU staining quantified SOX2’s impact on proliferative activity. All data represent mean ± SD (triplicate experiments); analyzed by unpaired t-tests (ns: no significance, *P < 0.05, **P < 0.01, ***P < 0.001, ****P < 0.0001).

We also verified the expression of SOX2 in glioma tissues and cells. Based on the results of the RT-PCR, SOX2 is highly expressed in glioma tissues (**Figure 9A**), and its expression increases with tumor grade (**Figure 9B**). A high level of SOX2 expression was also observed in glioma cells (**Figure 9C**). These findings established SOX2 as a key oncogene in glioma and a prime candidate for a functional partner of ZFHX4. Therefore, we proceeded to investigate the precise regulatory interplay between them and the functional consequences of this interaction.

**Figure 10:**
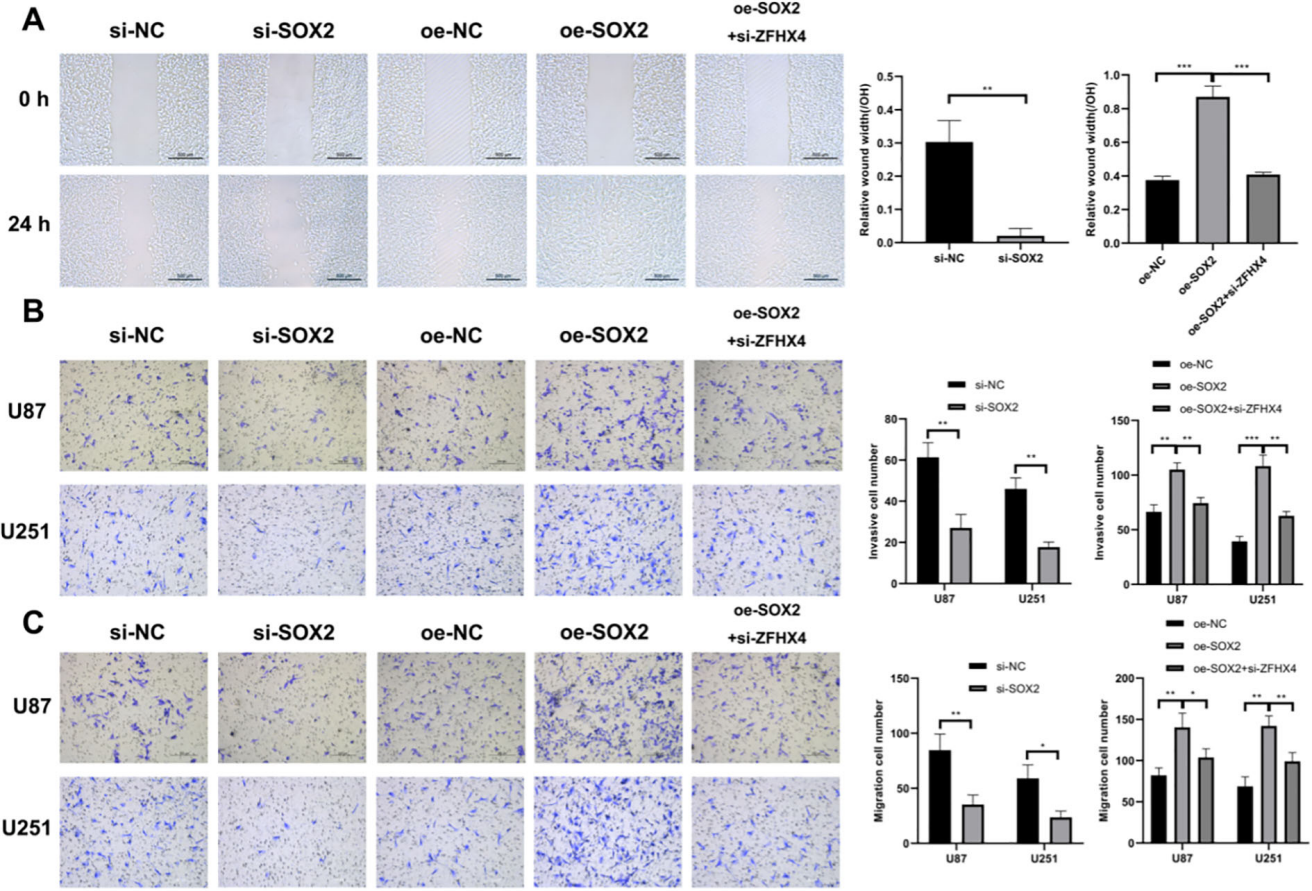
SOX2 promotes glioma cell invasion and migration. **(A)** Wound healing assays demonstrated SOX2 overexpression enhanced U251 cell migration, while knockdown suppressed it. **(B, C)** Transwell assays revealed: SOX2 overexpression increased invasion/migration in U87 and U251 cells; SOX2 knockdown attenuated invasive/migratory capacity. All data represent mean ± SD (triplicates); analyzed by unpaired t-tests (*P < 0.05, **P < 0.01, ***P < 0.001).

We knocked down SOX2 expression using siRNA and overexpressed it via plasmid in glioma cells in order to ascertain the role of SOX2 in glioma. The knockdown as well as the overexpression of SOX2 were both successfully verified. In U87 and U251 cells, si- SOX2 and oe- SOX2 respectively reduced the mRNA expression of SOX2 by more than 65% and overexpressed it by more than 3 times. (**Figures 9D, E**). Functional assays demonstrated that SOX2 silencing inhibited proliferation of glioma cells, whereas increased SOX2 expression enhanced proliferation of glioma cells by colony formation assay (**Figure 9F**), CCK-8 assay (**Figure 9G**), and EdU assay (**Figure 9H**). The knockdown of SOX2 significantly reduced glioma cell migration and invasion in wound healing assays (**Figure 10A**), transwell assays (**Figures 10B, C**). In contrast to cells in the control group, cells overexpressing SOX2 showed greater migration and invasion.

**Figure 11:**
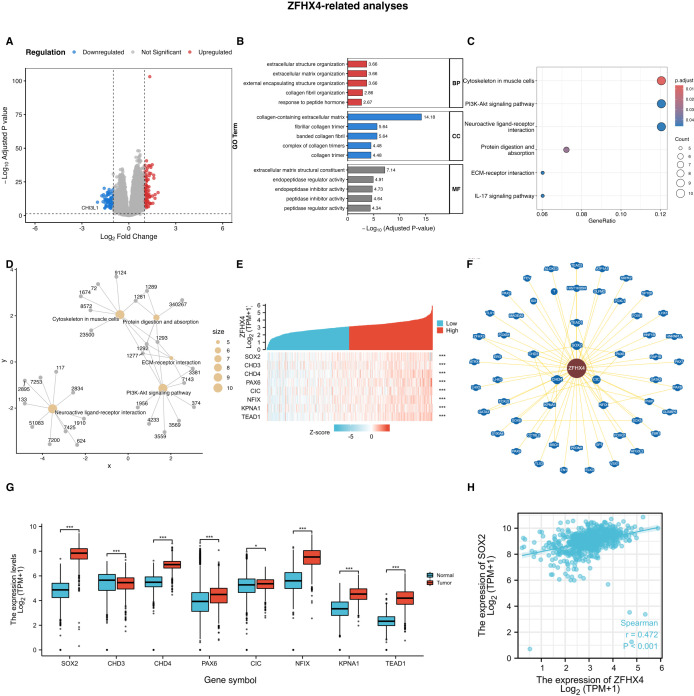
Reciprocal regulation between ZFHX4 and SOX2. **(A)** Co-immunoprecipitation (Co-IP) confirmed physical interaction between SOX2 and ZFHX4 in glioma cells. **(B, C)** ZFHX4 knockdown/overexpression significantly altered SOX2 expression (RT-qPCR/Western blotting). **(D, E)** SOX2 perturbation reciprocally modulated ZFHX4 levels. All data represent mean ± SD (triplicate experiments); analyzed by unpaired t-tests (*P < 0.05, **P < 0.01).

We confirmed that SOX2 and ZFHX4 can bind to each other in glioma cells by co-immunoprecipitation (**Figure 11A**). Following the knockdown of ZFHX4 and the overexpression of ZFHX4, there were substantial changes in the expression of SOX2 in glioma cells as determined by RT-PCR (**Figure 11B**) and Western Blot (**Figure 11C**) analysis. The results demonstrated that SOX2 expression decreased and increased in glioma cells following knockdown and overexpression of ZFHX4. We also detected ZFHX4 expression in glioma cells that overexpressed and knocked down SOX2. To determine if this regulation was unidirectional, we performed the reverse experiment and found that knockdown or overexpression of SOX2 also resulted in a corresponding decrease or increase in ZFHX4 expression, respectively (**Figures 11D, E**). This indicates a mutual, positive regulatory relationship between ZFHX4 and SOX2. Furthermore, overexpression of SOX2 followed by knockdown of ZFHX4 reversed the malignant biological behavior of glioma cells, such as proliferation, migration and invasion (**Figures 10A–C**). In addition, the above findings further confirmed that ZFHX4 contributes significantly to modulating proliferation, invasion, and metastatic processes in glioma cells through SOX2.

**Figure 12:**
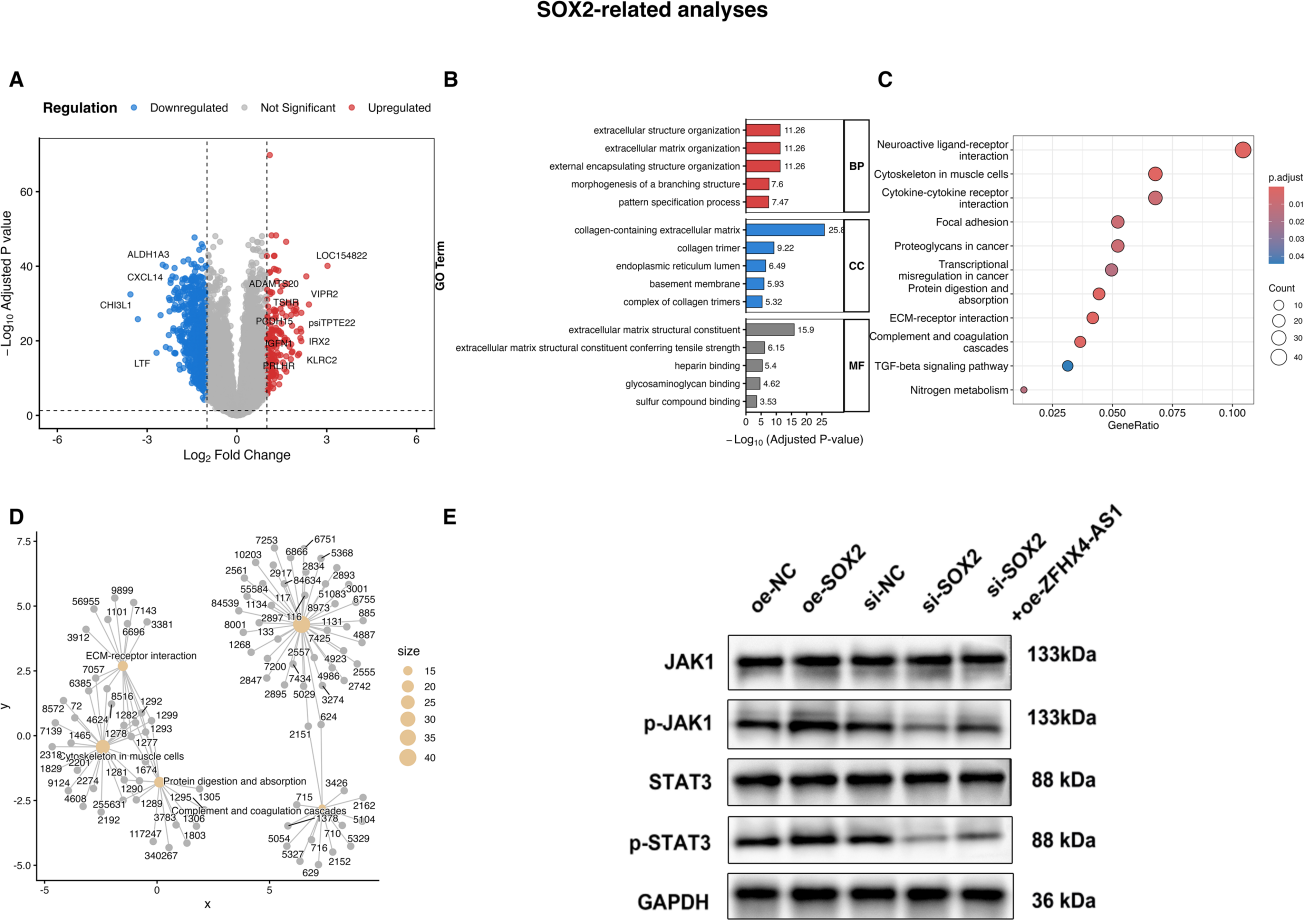
Differential expression and functional analysis of SOX2 (updated with FDR correction). **(A)** Volcano plot of DEGs stratified by SOX2 expression (low vs high). **(B)** GO enrichment analysis of SOX2-associated DEGs. **(C)** Bubble plot of KEGG pathway enrichment for SOX2-associated DEGs. **(D)** Gene-concept network illustrating the relationships between key DEGs and enriched KEGG pathways. **(E)** Validation: SOX2 knockdown suppressed JAK-STAT signaling, while ZFHX4-AS1 overexpression partially rescued this effect. *(Statistical thresholds: *P < 0.05, **P < 0.01, ***P < 0.001).

Based on analysis of DEGs in SOX2 high and low expression groups, we attempted to clarify the mechanism by which SOX2 regulates glioma development and occurrence. We screened 752 down-regulated genes and 189 up-regulated genes (**Figure 12A**). To understand the biological functions associated with these DEGs, we conducted Gene Ontology (GO) enrichment analysis. The analysis revealed that the DEGs were significantly enriched in biological processes related to the tumor microenvironment, such as extracellular structure organization and extracellular matrix organization. Key cellular component terms included collagen-containing extracellular matrix, while prominent molecular functions were associated with extracellular matrix structural constituent (**Figure 12B**). Furthermore, Kyoto Encyclopedia of Genes and Genomes (KEGG) pathway analysis highlighted several important signaling pathways, including Neuroactive ligand-receptor interaction, Cytokine-cytokine receptor interaction, and ECM-receptor interaction (**Figure 12C**). A gene-concept network was constructed to visualize the intricate connections between the key DEGs and these enriched pathways (**Figure 12D**). Given that the Cytokine-cytokine receptor interaction pathway is a critical upstream regulator of JAK-STAT signaling, we next sought to validate the activation of this key oncogenic pathway. We found that the phosphorylation ratios of JAK1 and STAT3 were altered upon both knockdown and overexpression of SOX2, and that overexpression of ZFHX4-AS1 after knockdown of SOX2 partially rescued these changes (**Figure 12E**).

## 4 Discussion

Our study elucidates a comprehensive oncogenic pathway in glioma initiated by the lncRNA ZFHX4-AS1. Numerous lncRNAs have emerged as valuable diagnostic and therapeutic indicators in cancer (24), including glioma. Building upon our preliminary findings that first identified a linear ZFHX4-AS1/ZFHX4/SOX2 axis (23), the present work provides a more complete mechanistic picture. We have now identified a critical ZFHX4/SOX2 positive feedback loop that amplifies the oncogenic signal, and have demonstrated that this entire axis ultimately converges on the activation of the JAK-STAT pathway.

To clarify the novelty of this study relative to our 2023 preprint (“Long non-coding RNA ZFHX4-AS1 Upregulates the Neural Differentiation-related Gene ZFHX4 and thereby Promotes SOX2 Expression to Accelerate Glioma Progression”), we provide a side-by-side comparison of the core findings. The preprint primarily established a unidirectional ZFHX4-AS1 → ZFHX4 → SOX2 axis that promotes glioma cell proliferation, migration, and invasion, supported by correlation and rescue assays. In the present study, we expand this model by identifying and validating a positive feedback loop between ZFHX4 and SOX2, revealing mutual transcriptional regulation rather than a single linear cascade. Moreover, the revised work further uncovers the JAK-STAT pathway as a downstream convergence point of this regulatory axis, supported by Western blot analyses of phosphorylated JAK1 and STAT3, and enrichment analyses of the updated DESeq2 dataset (with FDR correction). These additions establish a more complete mechanistic framework and significantly extend the biological and translational implications of our previous work.

### 4.1 ZFHX4-AS1 acts via its target ZFHX4 as an oncogenic driver in glioma

Our analyses consistently demonstrated that ZFHX4-AS1 functions as an oncogene in glioma. This conclusion is supported by both our bioinformatics analysis and direct experimental validation, which showed that ZFHX4-AS1 is significantly overexpressed in glioma tissues, and its high expression is associated with poor patient prognosis and higher tumor malignancy. To understand its mechanism, we investigated its regulation of the adjacent gene, ZFHX4. ZFHX4 has a known role in nerve and muscle differentiation and was previously identified as a regulatory factor linking the chromatin-remodeling NuRD complex to the glioblastoma tumor-initiating cell state (25, 26). Our work confirms its role as an oncogene in glioma and establishes that ZFHX4-AS1 positively regulates its expression in a unidirectional manner, thereby modulating the malignant behavior of glioma cells.

### 4.2 The ZFHX4/SOX2 positive feedback loop amplifies the oncogenic signal

A key mechanistic insight from this study is the identification of the stemness factor SOX2 as a critical functional partner of ZFHX4. The role of SOX2 in promoting cancer cell characteristics is well-established, and its expression in gliomas is known to correlate positively with the grade of malignancy (27, 28). Beyond a simple linear relationship, we uncovered a positive feedback loop where ZFHX4 and SOX2, which physically interact, mutually reinforce each other’s expression. In cancer biology, such loops act as potent signal amplifiers that create robust, self-sustaining oncogenic states. This ZFHX4/SOX2 regulatory circuit likely serves to amplify and stabilize the pro-malignancy signals initiated by ZFHX4-AS1, effectively “locking” glioma cells into a highly aggressive phenotype and offering a more nuanced understanding of glioma’s relentless progression.

### 4.3 Limitations and future directions

While our study comprehensively delineates this oncogenic pathway, we acknowledge several limitations that also open important avenues for future research. On a mechanistic level, our claim of cis-regulation is based on strong correlative evidence, but direct confirmation using techniques such as luciferase reporter assays is warranted. Similarly, while our data strongly supports the ZFHX4/SOX2 feedback loop, more intricate rescue experiments would provide more definitive proof. The functional necessity of the downstream JAK-STAT pathway could also be more definitively confirmed using specific pharmacological inhibitors.

Our experimental models and their translational relevance also present certain limitations. Regarding our clinical samples, while the in-house cohort of 30 patients was crucial for validating gene expression levels, survival data for this specific cohort was unavailable due to the anonymized nature of these pre-existing specimens. Consequently, our prognostic claims rely on the larger, well-annotated TCGA dataset. Furthermore, our *in vitro* findings must be interpreted with caution, as they are derived from relatively homogeneous cell lines that may not fully capture the complexity of glioma, a notoriously heterogeneous disease. Methodologically, we also acknowledge that we cannot completely exclude the potential for off-target effects from the siRNAs used. While the cell lines were obtained from a reputable commercial source, in-house STR profiling was not performed. Finally, our *in vivo* study was focused on overexpression models; future studies utilizing knockdown models and histological analysis would provide critical complementary evidence.

Despite these limitations, this study highlights the therapeutic potential of targeting this pathway. The ZFHX4-AS1/ZFHX4/SOX2 axis represents a promising set of targets for glioma intervention. Future investigations could therefore focus on developing novel therapeutic strategies, such as inhibitors against the ZFHX4-SOX2 interaction or exploring whether targeting the downstream JAK-STAT pathway is an effective approach in gliomas characterized by high ZFHX4-AS1 expression.

Additionally, future work should directly interrogate the mechanistic link between the ZFHX4/SOX2 axis and JAK-STAT activation. For instance, chromatin immunoprecipitation sequencing (ChIP-seq) or promoter-luciferase assays could determine whether SOX2 binds directly to the promoter regions of JAK or STAT family genes. Furthermore, pharmacological rescue experiments using specific JAK or STAT inhibitors could help establish the functional necessity of this pathway in mediating the oncogenic effects of the ZFHX4-AS1/ZFHX4/SOX2 loop. Such studies would provide causal evidence for the convergence of this axis on JAK-STAT signaling and strengthen the translational rationale for pathway-targeted therapy in glioma.

## 5 Conclusions

In conclusion, our study provides a comprehensive mechanistic model for the role of ZFHX4-AS1 in glioma. We establish that the lncRNA ZFHX4-AS1 initiates an oncogenic signal by upregulating its neighboring gene, ZFHX4. This signal is then powerfully amplified by a previously uncharacterized ZFHX4/SOX2 positive feedback loop, which serves to lock the cells in a malignant state. Ultimately, we demonstrate that this entire axis converges on the activation of the downstream JAK-STAT signaling pathway. By elucidating this complete ZFHX4-AS1/ZFHX4/SOX2/JAK-STAT pathway, our work moves beyond a simple linear model to provide a deeper understanding of glioma pathogenesis, identifying a more refined set of potential therapeutic targets for intervention.

## Data Availability

The datasets presented in this study can be found in online repositories. The names of the repository/repositories and accession number(s) can be found in the article/supplementary material. The underlying data for our manuscript are publicly available at the following link: https://zenodo.org/records/16790460.
